# Supplementary material for: Analysis of Phlebotomine sandflies in Laos from 2014–2024: Inventory, description of a new species, screening for Leishmania and detection of Trypanosoma
Source: PLoS Negl Trop Dis. 2026 Jan 2;20(1):e0013641. doi: 10.1371/journal.pntd.0013641 (PMC12795457; doi:10.1371/journal.pntd.0013641)
Supplement: S1 Text — (DOC) [file pntd.0013641.s001.doc]

**S1 Text: Technical analysis of the Trypanosoma phylogenetic tree**

1. **Sequences from NCBI used in this study**

Table A: Metadata of selected sequences included in this analysis

| Accession | Trypanosome | Host | Host_group | Geolocation |
| --- | --- | --- | --- | --- |
| AB175622 | Trypanosoma grosi | Apodemus agrarius | Rodents | Russia: Vladivostok |
| AB175624 | Trypanosoma grosi | Apodemus speciosus speciosus | Rodents | Japan: Aomori, Takko |
| AB175625 | Trypanosoma otospermophili | Spermophilus richardsonii | Rodents | Mongolian |
| AB175626 | Trypanosoma kuseli | Pteromys volans | Rodents | Mongolian |
| AB190228 | Trypanosoma otospermophili | Spermophilus columbianus | Rodents | Mongolian |
| AF362821 | Trypanosoma cruzi | Triatomine_Rhodnius brethesi | Insects | Brazil: Amazon basin |
| AF362827 | Trypanosoma cruzi | Cattle | Ungulates | Brasil |
| AY230234 | Trypanosoma rangeli | Triatomine_Rhodnius prolixus | Insects | Colombia |
| AY230236 | Trypanosoma rangeli | Homo sapiens | Human | Honduras |
| AY230237 | Trypanosoma rangeli | Homo sapiens | Human | Venezuela |
| AY230238 | Trypanosoma rangeli | Triatomine_Rhodnius prolixus | Insects | Venezuela |
| AY491772 | Trypanosoma rangeli | Homo sapiens | Human | El Salvador |
| AY491773 | Trypanosoma rangeli | Homo sapiens | Human | Panama |
| AY491774 | Trypanosoma rangeli | Echimys dasythrix | Rodents | Brazil |
| AY773714 | Trypanosoma sp. D30 | Deer | Ungulates | Germany |
| AY959322 | Trypanosoma avium | Metacnephia | Insects | Finland |
| EU861192 | Trypanosoma lewisi | Rattus norvegicus | Rodents | China: Baise of Guangxi |
| FJ555667 | Trypanosoma cruzi | Canis familiaris | Dog | Brazil: Mato Grosso do Sul |
| FJ555668 | Trypanosoma cruzi | Oxymycterus sp. | Rodents | Brazil: Para |
| FN677342 | Leishmania major | Homo sapiens | Human | Tunisia |
| GQ258718 | Trypanosoma cruzi | Triatomine_Rhodnius neglectus | Insects | Brazil: Sao Luiz, MA |
| JN673391 | Trypanosoma brucei | Crocuta crocuta (hyaena) | Carnivores | Tanzania: Serengeti National Park |
| JN673395 | Trypanosoma theileri | Kobus vardonii (puku) | Ungulates | Zambia: Musalangu Game Management Area |
| JN673398 | Trypanosoma sp. Z3206 | Hippopotamus amphibius (hippopotamus) | Ungulates | Zambia: Lumimba Game Management Area |
| JX178172 | Trypanosoma sp. PJH-2013a | Odocoileus virginianus | Ungulates | USA: Zapata Co., TX |
| JX178174 | Trypanosoma cf. cervi PJH-2013 | Odocoileus virginianus | Ungulates | USA: Zapata Co., TX |
| JX178178 | Trypanosoma sp. PJH-2013b | Cervus elaphus canadensis | Ungulates | USA: Minnesota |
| JX178179 | Trypanosoma cf. cervi PJH-2013 | Cervus elaphus canadensis | Ungulates | USA: Minnesota |
| JX443482 | Trypanosoma cf. avium TRM-2012 | Andropadus nigriceps | Bird | USA |
| JX853184 | Trypanosoma sp. PJH-2013b | Cervus elaphus canadensis (Elk) | Ungulates | USA |
| JX910374 | Trypanosoma congolense | Cattle | Ungulates | Burkina Faso |
| KJ467211 | Trypanosoma sp. AP-2014 | Sandfly | Insects | Thailand |
| LC339505 | Trypanosoma nabiasi | Sandfly_Phlebotomus perniciosus | Insects | Spain:Madrid |
| LC339506 | Trypanosoma nabiasi | Sandfly_Phlebotomus perniciosus | Insects | Spain:Madrid |
| LC339508 | Trypanosoma nabiasi | Sandfly_Phlebotomus perniciosus | Insects | Spain:Madrid |
| LC440405 | Trypanosoma theileri | Bos taurus, Cattle (#128) | Ungulates | Mongolia:Selenge, Tushig |
| LC492117 | Trypanosoma sp. SDNK48 | Equus asinus | Ungulates | Sudan:Khartoum State, West Omdurman |
| LC492119 | Trypanosoma sp. SDNK93 | Equus asinus | Ungulates | Sudan:Khartoum State, West Omdurman |
| LC492121 | Trypanosoma sp. SDNK9 | Equus asinus | Ungulates | Sudan:Khartoum State, West Omdurman |
| LC492122 | Trypanosoma sp. SDNK92 | Equus asinus | Ungulates | Sudan:Khartoum State, West Omdurman |
| LC492125 | Trypanosoma congolense type Savannah | Equus asinus | Ungulates | Sudan:Khartoum State, West Omdurman |
| LC521917 | Trypanosoma sp. | Capra hircus | Ungulates | Philippines: Cebu |
| LC546922 | Trypanosoma sp. | Capra hircus | Ungulates | Philippines: Bohol state, Ubay city |
| LC546941 | Trypanosoma sp. | Equus asinus | Ungulates | Sudan: Atbara, River Nile State |
| LC589621 | Trypanosoma sp. Tz155 | Equus caballus | Ungulates | Paraguay:Cordillera |
| LC589622 | Trypanosoma sp. Tz226 | Equus caballus | Ungulates | Paraguay:Guaira |
| LC589623 | Trypanosoma sp. Tz298 | Equus caballus | Ungulates | Paraguay:Caaguazu |
| LC589624 | Trypanosoma sp. Tz353 | Equus caballus | Ungulates | Paraguay:Central |
| LC589625 | Trypanosoma sp. Tz406 | Equus caballus | Ungulates | Paraguay:Cenral |
| MN121258 | Trypanosoma evansi | Rusa timorensis | Ungulates | Thailand: Krabi |
| OL332783 | Trypanosoma sp. | Sandfly_Sergentomyia sp. | Insects | Thailand: Satun |
| OL332784 | Trypanosoma sp. | Sandfly_Idiophlebotomus asperulus | Insects | Thailand: Satun |
| OL332785 | Trypanosoma sp. | Sandfly_Sergentomyia khawi | Insects | Thailand: Songkhla |
| OL332786 | Trypanosoma sp. | Sandfly_Sergentomyia indica | Insects | Thailand: Songkhla |
| OL332787 | Trypanosoma sp. | Sandfly_Sergentomyia khawi | Insects | Thailand: Songkhla |
| OL332788 | Trypanosoma sp. | Sandfly_Idiophlebotomus asperulus | Insects | Thailand: Phang-Nga |
| OL332789 | Trypanosoma sp. | Sandfly_Sergentomyia sp. | Insects | Thailand: Satun |
| OL332790 | Trypanosoma sp. | Sandfly_Sergentomyia barraudi | Insects | Thailand: Satun |
| ON731777 | Trypanosoma sp. | Sandfly_Sergentomyia squamipleuris | Insects | Kenya: Laisamis, Marsabit County |
| OP297201 | Trypanosoma grosi | Crocidura sp. | Shrews | South Korea |
| OQ726218 | Trypanosoma lewisi | Homo sapiens | Human | India |
| OR973749 | Trypanosoma theileri | Cattle | Ungulates | Cote d'Ivoire |
| OR973775 | Trypanosoma theileri | Cattle | Ungulates | Cote d'Ivoire |
| PV034524 | Trypanosoma sp. SF22-Ex02 | Sandfly_Idiophlebotomus longiforceps |  | Laos: Viengphoukha, Luangnamtha Province |
| PV034525 | Trypanosoma sp. SF22-Ex243 | Sandfly_Idiophlebotomus longiforceps |  | Laos: Kasi, Vientiane Province |
| PV034526 | Trypanosoma sp. SF22-Ex446 | Sandfly_Chinius eunicegalatiae |  | Laos: Vientiane Province |
| PV034527 | Trypanosoma sp. SF22-Ex597 | Sandfly_Chinius eunicegalatiae |  | Laos: Vientiane Province |
| PV034528 | Trypanosoma sp. SF22-Ex598 | Sandfly_Chinius eunicegalatiae |  | Laos: Vientiane Province |
| PV034529 | Trypanosoma sp. SF22-EX599 | Sandfly_Chinius eunicegalatiae |  | Laos: Vientiane Province |
| PV034530 | Trypanosoma sp. SF22-EX600 | Sandfly_Chinius eunicegalatiae |  | Laos: Vientiane Province |
| PV034531 | Trypanosoma sp. SF22-EX601 | Sandfly_Chinius eunicegalatiae |  | Laos: Vientiane Province |
| PV034532 | Trypanosoma sp. SF22-EX602 | Sandfly_Chinius eunicegalatiae |  | Laos: Vientiane Province |
| PV034533 | Trypanosoma sp. SF22-EX606 | Sandfly_Chinius eunicegalatiae |  | Laos: Vientiane Province |
| PV034534 | Trypanosoma sp. SF22-EX607 | Sandfly_Chinius eunicegalatiae |  | Laos: Vientiane Province |
| PV034535 | Trypanosoma sp. SF22-EX608 | Sandfly_Chinius eunicegalatiae |  | Laos: Vientiane Province |
| PV034536 | Trypanosoma sp. SF22-EX610 | Sandfly_Chinius eunicegalatiae |  | Laos: Vientiane Province |
| PV034537 | Trypanosoma sp. SF22-EX611 | Sandfly_Chinius eunicegalatiae |  | Laos: Vientiane Province |
| PV034538 | Trypanosoma sp. SF22-EX617 | Sandfly_Chinius eunicegalatiae |  | Laos: Vientiane Province |
| PV034539 | Trypanosoma sp. SF22-EX618 | Sandfly_Chinius eunicegalatiae |  | Laos: Vientiane Province |
| PV034540 | Trypanosoma sp. SF22-EX619 | Sandfly_Chinius eunicegalatiae |  | Laos: Vientiane Province |
| PV034541 | Trypanosoma sp. SF22-EX620 | Sandfly_Chinius eunicegalatiae |  | Laos: Vientiane Province |
| PV034542 | Trypanosoma sp. SF22-Ex622 | Sandfly_Chinius eunicegalatiae |  | Laos: Vientiane Province |
| PV034543 | Trypanosoma sp. SF22-Ex623 | Sandfly_Chinius eunicegalatiae |  | Laos: Vientiane Province |
| PV034544 | Trypanosoma sp. SF22-Ex628 | Sandfly_Chinius eunicegalatiae |  | Laos: Vientiane Province |
| PV034545 | Trypanosoma sp. SF22-Ex630 | Sandfly_Chinius eunicegalatiae |  | Laos: Vientiane Province |
| PV034546 | Trypanosoma sp. SF22-Ex631 | Sandfly_Chinius eunicegalatiae |  | Laos: Vientiane Province |
| PV034547 | Trypanosoma sp. SF22-Ex632 | Sandfly_Chinius eunicegalatiae |  | Laos: Vientiane Province |

1. **Alignment and curation methods**
   1. **Alignment using MAFFT v7.505**

A two-step strategy was used using MAFFT v7.505 alignment following Katoh et al. (2002). first, a pre-aligned set of full-length reference sequences; described as a backbone multiple sequence alignment (MSA); was created from reference sequences; then, new sequences were added to this backbone without disrupting the original alignment structure.

- - 1. **Step 1: Retrieval and alignment of MSA**

Ten *Trypanosoma* sequences containing genes: IGS, 18S rRNA, 5.8S rRNA, 28S rRNA and ITS1-6 were downloaded from NCBI GenBank to create a pre-aligned set of full-length reference sequences (Fig.A). The reference sequences were aligned using both Geneious Prime 2025.1.2 for manual observation and MAFFT v7.505 with the following command to create a backbone multiple sequence alignment (MSA):

mafft --maxiterate 1000 --localpair 10_Sequences_NCB_Reference.fasta > backbone_msa.fasta

This alignment used the –localpair (linsi) option for high accuracy and --maxiterate 1000 to ensure robust alignment of conserved and variable regions, following Katoh et al. (2002). The resulting backbone MSA was used in subsequent steps to incorporate other sequences, as described below. Results were import into Geneious Prime v2025.1.2 for visualisation as Fig. A and B below:


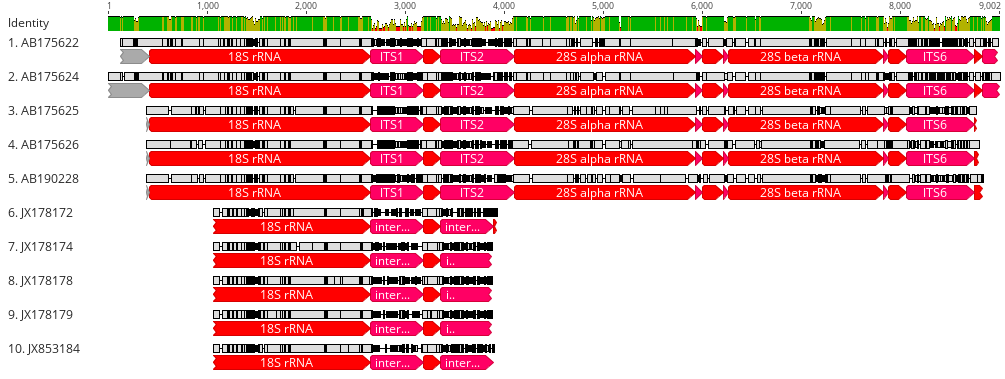


**ITS1+5.8S region**

Fig A: The backbone MSA (a pre-aligned set of sequences) using Geneious Prime.


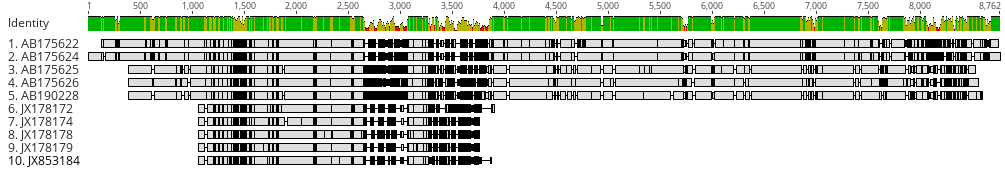


**ITS1+5.8S region**

Fig B: The backbone MSA (a pre-aligned set of sequences) using mafft with options --maxiterate 1000 --localpair.

- - 1. **Step 2: Adding new sequences to the backbone MSA**

Additional ITS1 and 5.8S rRNA sequences, including 24 sequences generated in this study from sandflies and other 53 reference sequences from NCBI GenBank, were aligned to the backbone MSA created in Step 1. The alignment was performed using MAFFT v7.505 with the --addfragments option to preserve the structure of the reference alignment, following Katoh et al. (2002) (visualization in Fig. C):

mafft --multipair --addfragments new_sequences.fasta backbone_msa.fasta > mafft_aligned.fasta


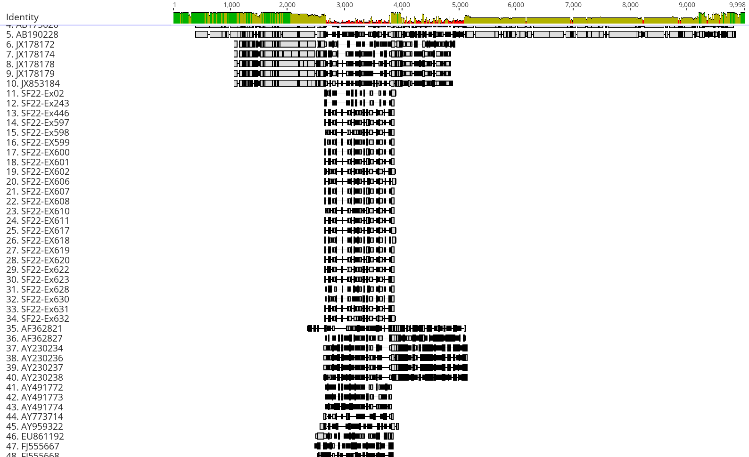


**ITS1+5.8S region**

Fig C: Alignment of additional sequences to the backbone MSA from step1 using mafft with options --multipair --addfragments.

- 1. **Alignment using MUSCLE and CLUSTALW algorithms in MEGA v12 software**

Selected aligned region of ITS1+5.8S from MAFFT alignment above was also tested for aligning using MUSCLE and CLUSTALW algorithms in MEGA v12 software. The resulting alignments were imported into Geneious Prime 2025.1.2 for visualization and manual curation.

We observed that all aligned methods confirmed high divergence between ITS1+5.8s sequences from this study and those available in GenBank database (Fig. A-E).


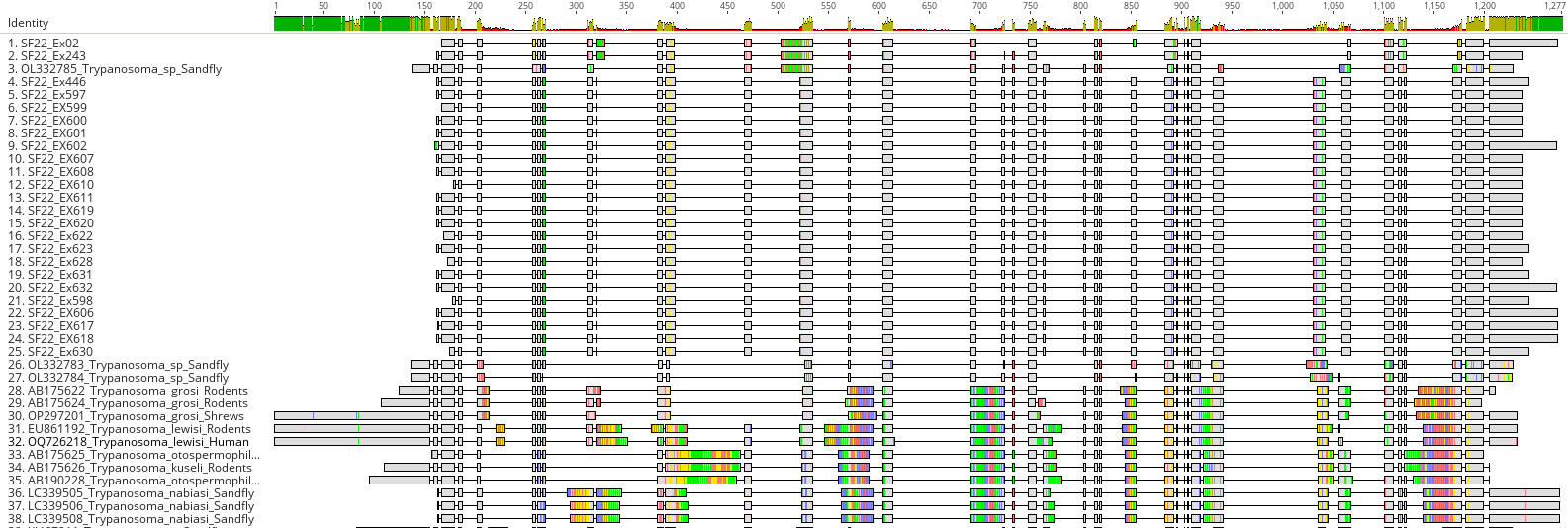


Fig D: Alignment of ITS1+5.8S sequences using MUSCLE algorithm.


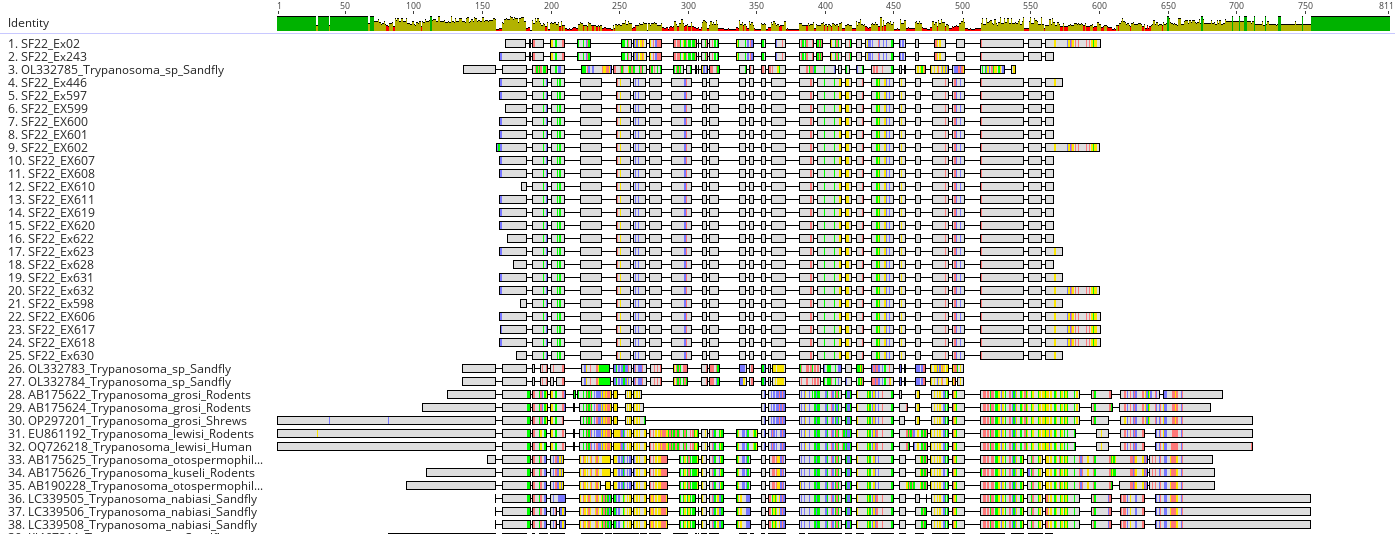


Fig E: Alignment of ITS1+5.8s sequences using CLUSTALW algorithm.

Ambiguous positions and gaps were inspected and resolved to ensure alignment quality, with poorly aligned regions were trimmed using BMGE v1.12 with default parameters, following Criscuolo et al. (2010).

1. **Tree construction**
   1. **IQ-TREE server**
2. Maximum likelihood phylogenetic tree analysis was conducted using IQ-TREE server (<http://iqtree.cibiv.univie.ac.at/>), with the substitution model automatically selected. The following command was used:

iqtree -s curated_msa.fasta -m TEST -bb 1000 -alrt 1000 -abayes

- 1. **MEGA v12 software**

In parallel with IQ-TREE server, the curated alignment was also analysed in MEGA v12. The model selections were based on the Bayesian Information Criterion (BIC) in the ModelTest tool.


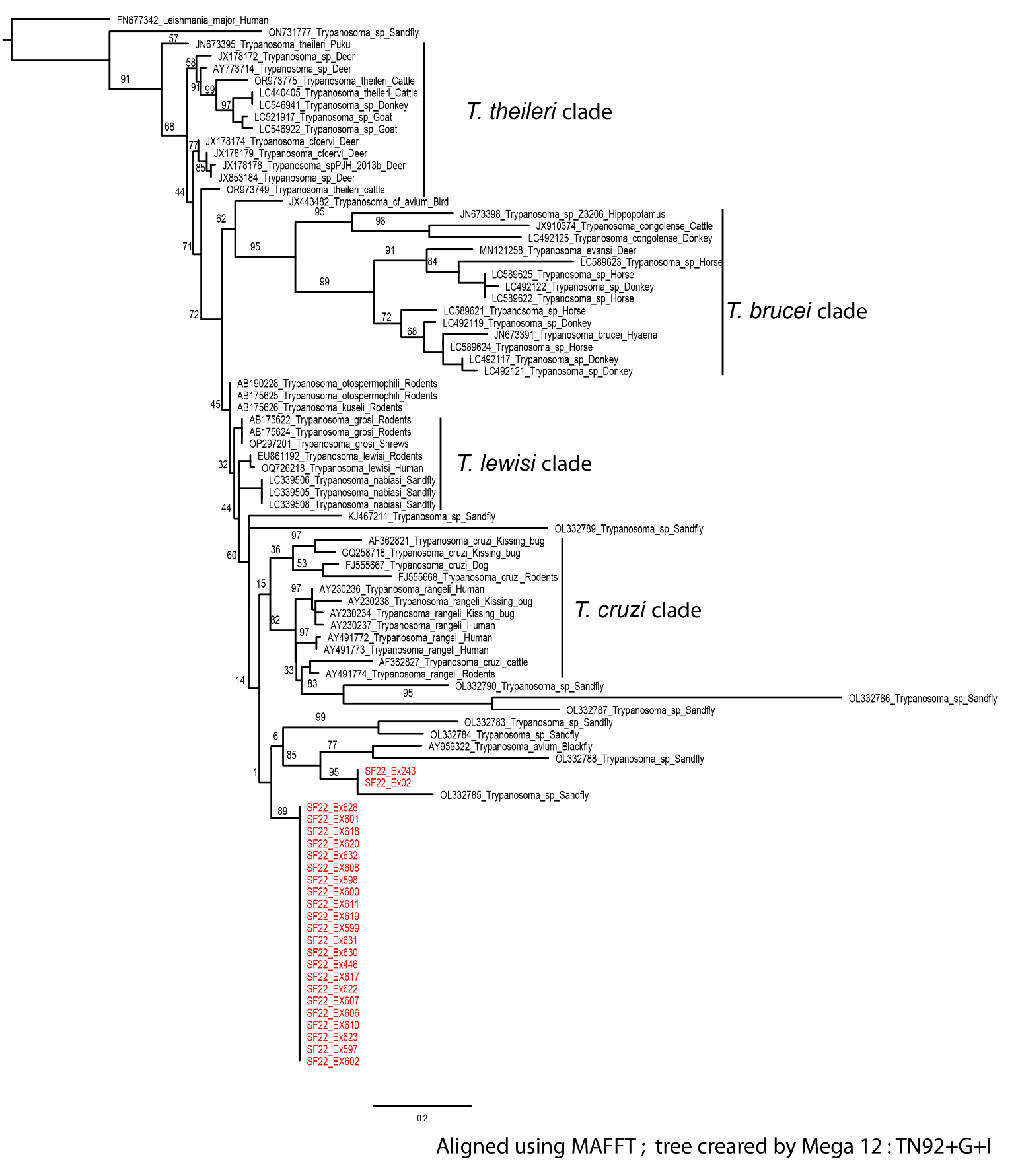


Fig F: Maximum likelihood phylogenetic tree constructed from *Trypanosoma* partial ITS1 and 5.8s region. Sequences were aligned using MAFFT and poorly aligned regions were trimmed using BMGE v1.12. The tree was constructed using MEGA v12 software with the TN92+G+I based on the Bayesian Information Criterion (BIC) in the ModelTest tool. The numbers on the branches represent the bootstrap values (%) derived from adaptive bootstrap replicates in MEGA software. Sequences obtained from this study in Laos are highlighted in red. *Leishmania major* (FN677342) was used as the outgroup.


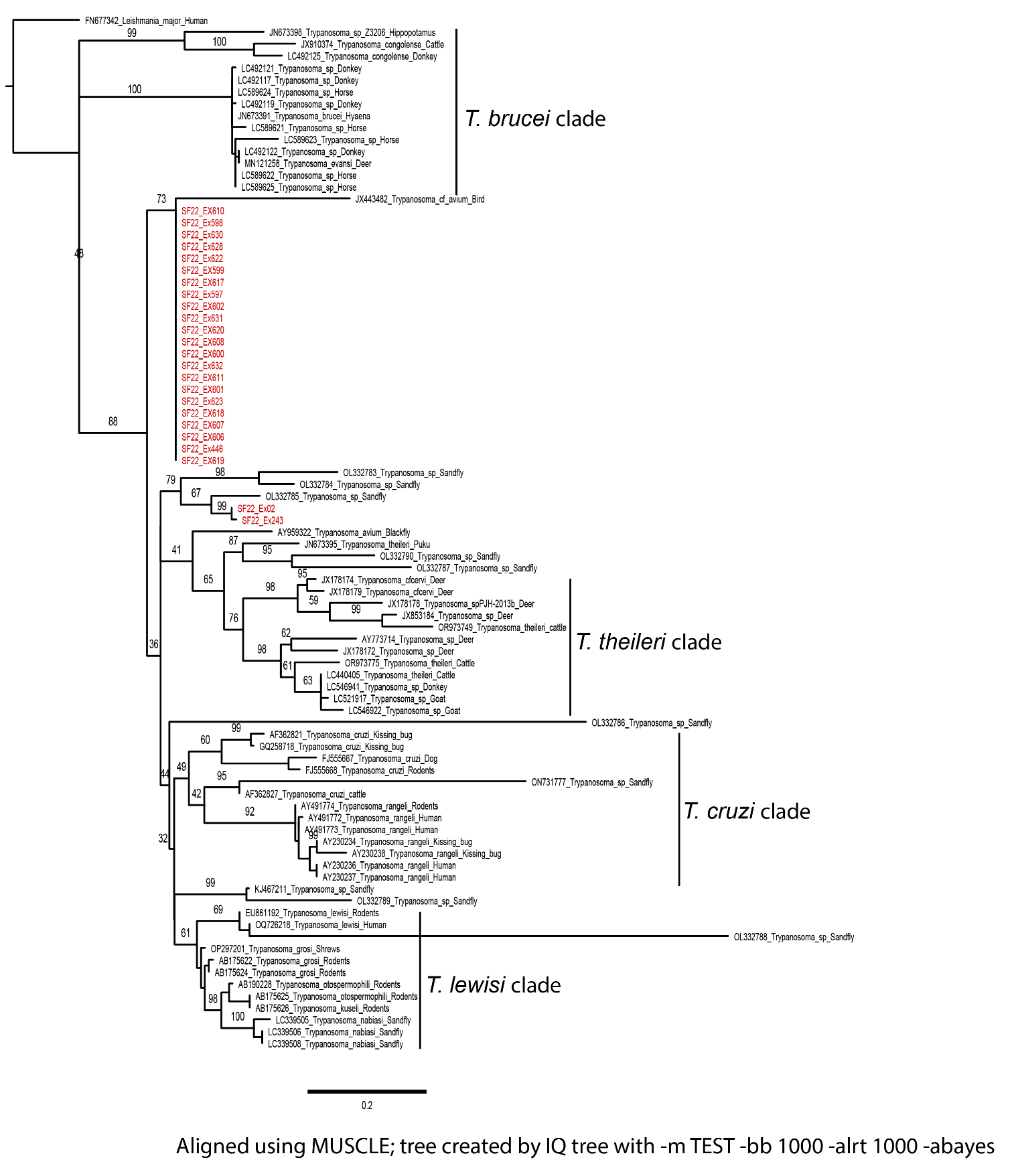


Fig G: Maximum likelihood phylogenetic tree constructed from Trypanosoma partial ITS1 and 5.8s region selected (Fig. C above). Sequences were realigned using MUSCLE and poorly aligned regions were trimmed using BMGE v1.12. The tree was constructed using IQ-TREE with model auto selected based on Bayesian Information Criterion (BIC). The numbers on the branches represent the bootstrap values (%) derived from 1000 replicates. Sequences obtained from this study in Laos are highlighted in red. *Leishmania* *major* (FN677342) was used as the outgroup.


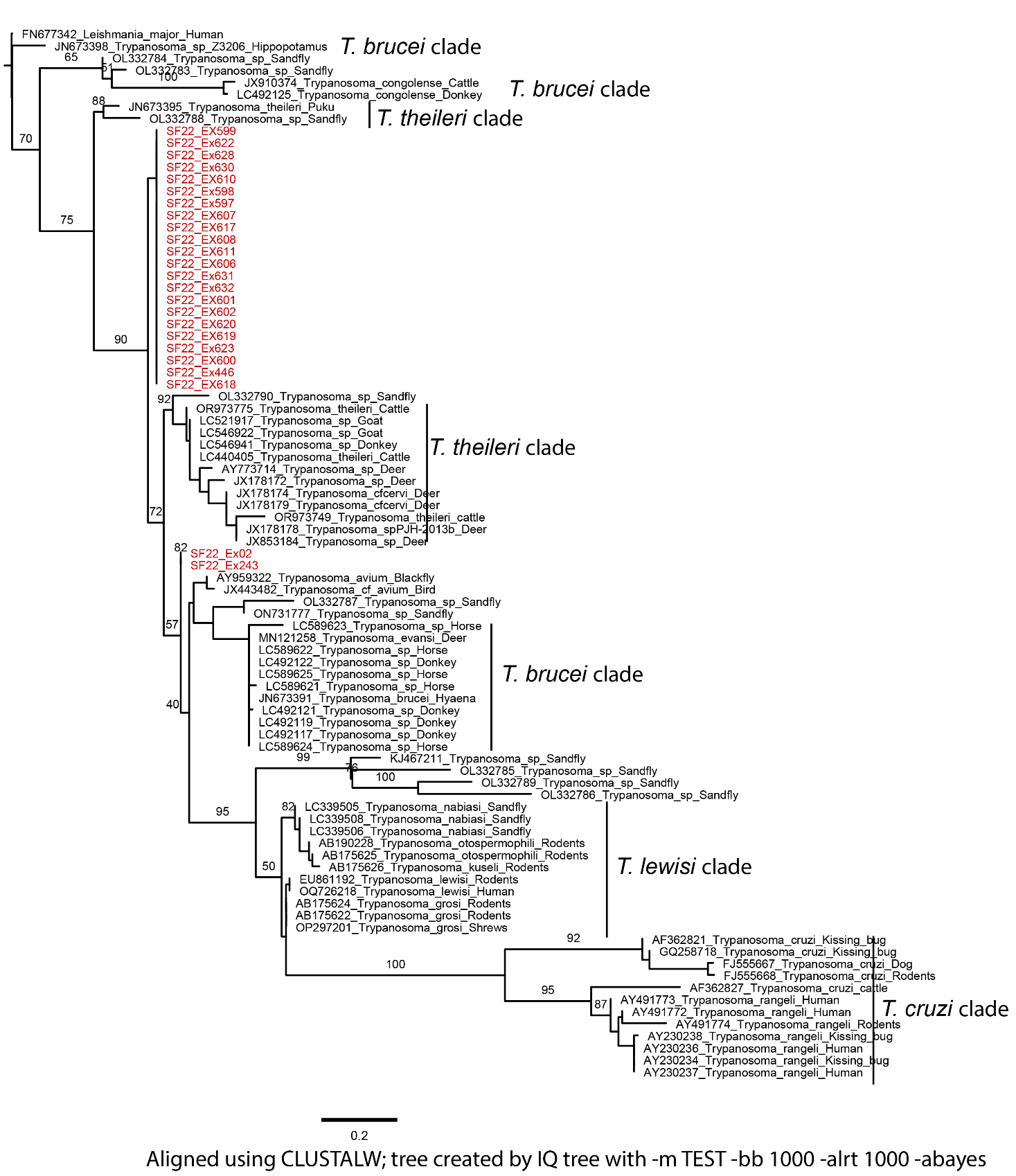


Fig H: Maximum likelihood phylogenetic tree constructed from Trypanosoma partial ITS1 and 5.8s region selected (Fig. C above). Sequences were realigned using CLUSTALW and poorly aligned regions were trimmed using BMGE v1.12. The tree was constructed using IQ-TREE with model auto selected based on Bayesian Information Criterion (BIC), The numbers on the branches represent the bootstrap values (%) derived from 1000 replicates. Sequences obtained from this study in Laos are highlighted in red. *Leishmania* *major* (FN677342) was used as the outgroup.

1. **Reference**

Katoh K, Misawa K, Kuma K, Miyata T. MAFFT: a novel method for rapid multiple sequence alignment based on fast Fourier transform. Nucleic Acids Res. 2002 Jul 15;30(14):3059-66. doi: 10.1093/nar/gkf436. PMID: 12136088; PMCID: PMC135756.

Criscuolo A, Gribaldo S. BMGE (Block Mapping and Gathering with Entropy): a new software for selection of phylogenetic informative regions from multiple sequence alignments. BMC Evol Biol. 2010;10:210. doi: 10.1186/1471-2148-10-210. PMID: 20626897; PMCID: PMC3017758.
