# Supplementary figures and images for: Analysis of Phlebotomine sandflies in Laos from 2014–2024: Inventory, description of a new species, screening for Leishmania and detection of Trypanosoma
Source: PLoS Negl Trop Dis. 2026 Jan 2;20(1):e0013641. doi: 10.1371/journal.pntd.0013641 (PMC12795457; doi:10.1371/journal.pntd.0013641)

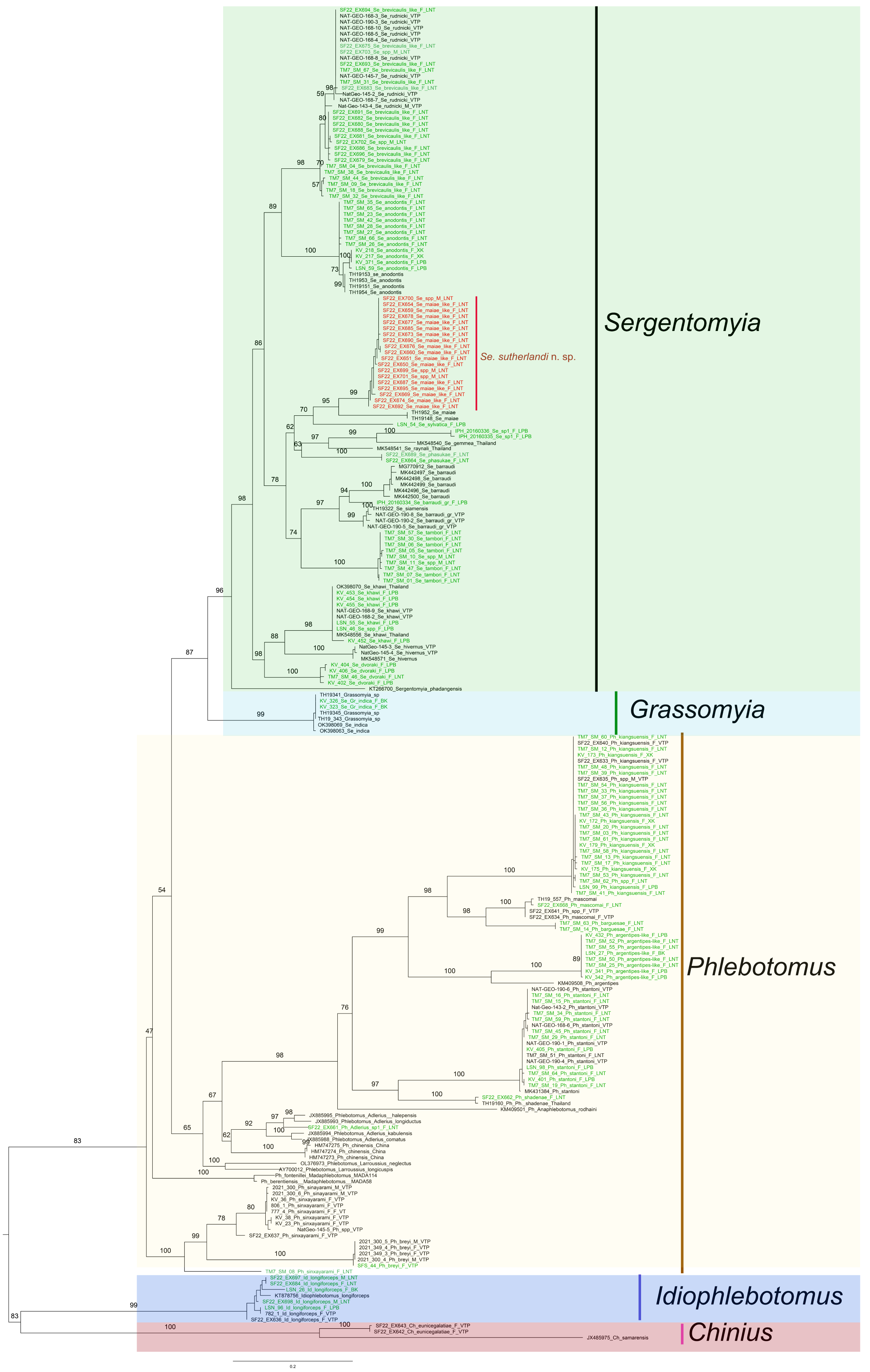

Supplement: S1 Fig — The tree was constructed using IQ-TREE with model auto selected based on Bayesian Information Criterion (BIC). The numbers on the branches represent the bootstrap values (%) derived from 1000 replicates (options: -m TEST -bb 1000 -alrt 1000 -abayes). Reference sequences of each species generated in previous studies and available in GenBank were selected and included in this analysis. Green color indicates species found in this study. New species that are closely related to Se. maiae are labelled in red color. (TIF) [file pntd.0013641.s006.tif]
