# Supplementary material for: Analysis of Phlebotomine sandflies in Laos from 2014–2024: Inventory, description of a new species, screening for Leishmania and detection of Trypanosoma
Source: PLoS Negl Trop Dis. 2026 Jan 2;20(1):e0013641. doi: 10.1371/journal.pntd.0013641 (PMC12795457; doi:10.1371/journal.pntd.0013641)
Supplement: Attachment — Submitted filename: Rebuttal_letter20250527.docx [file pntd.0013641.s007.docx]

**Response to Reviewers**

Dear Editors,

Please find below our response to reviewers, other changes were also added directly in the manuscript.

Best regards,

Khamsing Vongphayloth

**Reviewers' Comments:**

Reviewer's Responses to Questions

**Key Review Criteria Required for Acceptance?**

As you describe the new analyses required for acceptance, please consider the following:

**Methods:**

-Are the objectives of the study clearly articulated with a clear testable hypothesis stated?

-Is the study design appropriate to address the stated objectives?

-Is the population clearly described and appropriate for the hypothesis being tested?

-Is the sample size sufficient to ensure adequate power to address the hypothesis being tested?

-Were correct statistical analysis used to support conclusions?

-Are there concerns about ethical or regulatory requirements being met?

Reviewer #1: The manuscript "Analysis of Phlebotomine sandflies in Laos from 2014-2024: inventory, description of a new species, screening for Leishmania and detection of Trypanosoma" is very interesting as it provides data from a place where little is known about both on sandflies and sandfly-borne pathogens. The study aimed to report species diversity data and Leishmania detection among sandflies collected from seven provinces of Laos. The objectives of the study are clearly articulated and properly designed, based on their assumption that the bordering country to Laos, Thailand has reported autochthonous cases of leishmaniases. 3,857 sandflies from 25 species belonging to five genera were collected for nearly a decade (2014-2024) and identified morphologically and molecularly. They performed PCR-technique targetting the ITS1 region to screen for Leishmania in the sandfly samples and by direct nucleotide sequencing. The methods are properly described and the sample size of sandflies collected is suitable to address their hypothesis. The study does not need to perform statistical analysis. However, proper software were used for phylogenetic analysis.

Reviewer #2: Please see my General Comments.

Reviewer #3: The methods are okay, but the phylogenetics was poorly done. It's questionable whether the primer for blood-meal analysis were appropriate. The authors should be performing morphometric analysis on their sandflies.

Reviewer #4: The manuscript presents adequately described the objetives. Being a an ecological descriptive study sample size is not required.

**Results:**

-Does the analysis presented match the analysis plan?

-Are the results clearly and completely presented?

-Are the figures (Tables, Images) of sufficient quality for clarity?

Reviewer #1: The results are properly presented and match the analysis plan. The figures are of good quality.

Reviewer #2: Please see my General Comments.

Reviewer #3: Potentially interesting: the authors obtained 3 positive pools from 221 pools comprising a few thousand flies. It is a huge shame the blood meal analysis failed and I suggest the authors try again and do a full literature review of

The Tryp sp. tree was incomprehensible. There needs to be landmark trips to see what the species might be close to. I would suggest creating a rich species dense tree around all sequences associated with T. Rangel, which will include T. cruzi. Just about all these genomes have been sequenced so extracting the ITS should be easy. The issue is I can see a tree, full of Trypanosome sp. and I have no idea what it's saying. I recognise T. avium and of course T. rangel but thats about it. Thats not very informative of the species rich diversity of Tryps. Ultimately 3 tryps form a few thousand sandflies doesn't really jump out at me, but its interesting. If the blood meal analysis identified the specific vertebrates/mammals then that starts looking much more interesting. If the authors read the historic papers by Stevens et al and I would assume the genome trp trees after this (if they ever finished them), that is diversity of tryps you need for a macro-scale tryp tree and will help root the subsequent species specific tree: then a smaller tree correctly rooted from the macro-scale tree focusing around T. rangel and every sequence on Genbank therein. Do a series of Blasts via teh NCBI website - the entire sequences can be automatically downloaded by "cutting" the flanking loci of the hits. This will then need aligning (e.g. muscle5) and then building a tree via IQTREE. Make sure the alignment is correctly manually edited via appropriate software.

I can not comment on sandflies. I can't interpret the tree, albeit the methods are not state of the art, but thats relatively easy to correct. The authors should make their own investigation into this (guidance below) and <https://bioinformatics.stackexchange.com> may provide support.

Response:

We followed the valuable recommendations of the Reviewer. We retrieved more ITS1 sequences of *Trypanosoma sp*. from NCBI (a total of 63 sequences), aligned them using the ClustalW algorithm in MEGA11 software, and regenerated a new ML tree (Fig. 6).

Reviewer #4: Result are clearly described and in concordance with the objetives and methods described. All figures and tables are necesary.

**Conclusions:**

-Are the conclusions supported by the data presented?

-Are the limitations of analysis clearly described?

-Do the authors discuss how these data can be helpful to advance our understanding of the topic under study?

-Is public health relevance addressed?

Reviewer #1: Yes to all

Reviewer #2: Please see my General Comments.

Reviewer #3: The conclusions are interesting, but underwhelming.

Basically if the sandfly experts think the authors have an interesting observations on their new species I am happy to look at providing extensive revisions of the manuscript, but at present there are a lot of rough edges here. Again, if there were 24 decisive blood meal identifications the authors would be in a strong position to claim a paper at PLoS NTD

Reviewer #4: Conclussion are adequate to the objetive of the study.

**Editorial and Data Presentation Modifications?**

Use this section for editorial suggestions as well as relatively minor modifications of existing data that would enhance clarity. If the only modifications needed are minor and/or editorial, you may wish to recommend “Minor Revision” or “Accept”.

Reviewer #1: This is an interesting manuscript and provides data from a place where little is known about the diversity of the sandfly population. Although the aim of the study was also to detect the presence of Leishmania parasites based on the assumption that the bordering country, Thailand that has reported cases of leishmaniasis, the study contributes to the literature by showing the absence of Leishmania but the presence of Trypanosoma. I suggest it is interesting to publish the study.

Reviewer #2: Please see my General Comments.

Reviewer #3: The tryp tree labels are incomprehensible and need a graphics designer to help the authors. That tree is not just not publication standard, its review standard, again I cannot read some of the species labels they are so blurred.

Reviewer #4: A minor modification is suggested for lines 49-53 for be more clear.

Response:

We changed lines 49-53: from “Sandflies in Laos exhibit significant diversity and distribution across seven provinces, with species composition varying by habitat. Unknown *Trypanosoma* DNA was detected using the Leishmania PCR test. Studies should be conducted in wider geographical location with a higher number of traps per area in order to collect a greater number of sandflies to better define this vector and its pathogens.”

To “This study reveals a notable diversity of sandfly species across seven provinces in Laos, with the highest species richness observed in karstic cave environments. While no *Leishmania* DNA was detected, the unexpected identification of unknown *Trypanosoma* species in *Chinius eunicegalatiae* and *Idiophlebotomus longiforceps* suggests the potential presence of unrecognized trypanosomatid pathogens in the region. These findings underscore the need for broader geographic surveillance and increased sampling efforts to improve our understanding of sandfly ecology and the pathogens they may carry in Laos.”.

**Summary and General Comments:**

Use this section to provide overall comments, discuss strengths/weaknesses of the study, novelty, significance, general execution and scholarship. You may also include additional comments for the author, including concerns about dual publication, research ethics, or publication ethics. If requesting major revision, please articulate the new experiments that are needed.

Reviewer #1: This is an interesting manuscript and provides data from a place where little is known about the diversity of the sandfly population. Although the aim of the study was also to detect the presence of Leishmania parasites based on the assumption that the bordering country, Thailand that has reported cases of leishmaniasis, the study contributes to the literature by showing the absence of Leishmania in the region studied but the presence of Trypanosoma. Unfortunately, the authors did not go further to identify the Trypanosoma species.

Reviewer #2: Dear Editor,
In this study, Vongphayloth and colleagues investigated the diversity of phlebotomine sand flies in Laos. The manuscript presents a large-scale field study with a remarkable taxonomic effort, identifying 3,857 specimens belonging to 25 different phlebotomine sand fly species. In addition to classical morphological taxonomy, the authors employed a PCR-based method to amplify a fragment of the cytochrome b gene for molecular species confirmation. Through both approaches, they observed morphological and molecular differences suggesting the presence of a previously undescribed phlebotomine sand fly species. Consequently, they describe Sergentomyia sutherlandi n. sp., integrating both morphological and molecular traits. Finally, the authors attempted to detect Leishmania sp. DNA in the collected sand flies, which was unsuccessful, but they did report the presence of Trypanosoma sp. DNA in a few specimens.
I find this study highly valuable to the scientific community. Beyond describing a new species of phlebotomine sand fly, this study may provide genetic data for several species that are challenging to identify morphologically and could contribute to discussions on their taxonomic status.
While this version of the manuscript is well-written and presents solid research, I have some comments that the authors may find helpful in further improving and clarifying certain points.
Thank you for your general comments and overall positive feedback.

Introduction:
This section is consistent and well-structured.
Methods:
Sand fly cytochrome b gene database:
• Please cite the sequencing instrument used.

Response:

We added: “…for Sanger sequencing (Applied Biosystems™ 3500xL Genetic Analyzer).”
• Ensure that the collection site location is included in the GenBank features (i.e., geo_loc_name). This is an important detail often overlooked when submitting sequences to GenBank.

Response:

Yes, we included the site location in GenBank has been added.

Blood meal analysis:
• Did the authors include a PCR positive control for this analysis? Since no detection was reported (Results, line 353), I wonder whether this outcome was due to a late-stage blood meal that had already been digested (most likely) or a technical issue.

Response:

Yes, we included two positive controls in each series of PCR tests. We added in Methods : Bloodmeal analysis: “Human blood samples were used as positive controls.”.

Molecular detection of Leishmania and Trypanosoma species in sand flies:
• Was a PCR positive control included?

Response:

Yes, we included a *L. tropica* and *L. major* positive control and a negative control with every PCR run. This has been added to lines 205-206 (Methods).

Phylogenetic analysis of sand fly species and detected parasites:
• Please double-check the type of phylogenetic inference used by IQ-TREE. To the best of my knowledge, this tool employs maximum-likelihood inference rather than Bayesian inference.

Response:

In lines 220-222: changed to “Maximum likelihood phylogenetic analysis was carried out using IQ-TREE on the IQ-TREE web server (http://iqtree.cibiv.univie.ac.at/) using the approximate Bayes test with 1000 ultrafast bootstrap replicates.”
**Results:**
Construction of cyt-b database and phylogenetic analysis of sand flies:
• Lines 220–225: This detailed information seems excessive, as the number of sequences per species is already provided in Table S2.

Response:

We agreed to change lines 253-254 from: “A total of 141/150 samples from this study had the *cyt-b* gene successfully sequenced including *Ch. eunicegalatiae* (2)*, Id. longiforceps* (6), *Ph. barguesae* (2), *Ph. (Adlerius)* sp.1 (1), *Ph. kiangsuensis* (26), *Ph. mascomai* (3), *Ph. argentipes-like* (8), *Ph. shadenae* (1), *Ph. sinxayarami* (2), *Ph. stantoni* (12), *Se. anodontis* (12), *Se. barraudi* group (1), *Se. brevicaulis* (22), *Se. dvoraki* (4), *Se. khawi* (6), *Se. sutherlandi* n. sp. (19), *Se. phasukae* (2), *Se. sylvatica* (1), *Se. tambori* (9), and *Gr. indica* (2)”

To read: “A total of 141/150 samples from this study had the *cyt-b* gene successfully sequenced, including 20 species belonging to five genera (S2 Table).”

• Figure 2 (phylogenetic tree): Details on the criteria used to select sequences from GenBank would improve data interpretation for readers.

Response:

We changed Figure 2 to “Maximum likelihood phylogenetic tree constructed from 222 cyt-b sequences of 397 nt using the approximate Bayes test with 1000 bootstrap replicates. Reference sequences of each species generated in previous studies and available in GenBank were selected and included in this analysis. Green color indicates species found in this study. New species that are closely related to Se. maiae are labelled in red color. “n” indicates the number of sequences used for the analysis.”

o I recommend including a full-view tree (i.e., without collapsing nodes per species/groups) as a supplementary file. Please ensure that accession codes for all sequences are included.

Response:

We agree and have added a full-view tree as a supporting file (S1 Figure)

o Lines 240–242: What is the authors’ interpretation of the low pairwise distance between Se. rudnicki and Se. brevicaulis? Since these two species are collapsed into the Se. brevicaulis group clade, it is difficult to interpret this result. A full-view tree or a revised version showing the clade with nominal species might clarify this.

Response:

We interpreted these results and discussed in the discussion section in lines 463-476:

Sergentomyia brevicaulis is closely related to Se. rudnicki and Se. barraudi group in presenting a comb-like cibarial armature. Sergentomyia barraudi group could be separated from Se. rudnicki and Se. brevicaulis by their shorter antennae flagellomere 1, and cibarial sclerotized area forked anteriorly. Sergentomyia brevicaulis was originally described from Vietnam in 1962 in having cibarium with about 50 comb-liked teeth and one row of anterior teeth [16]. Later, Se. rudnicki was described from specimens from Malaysia in 1978 by females with cibarium exhibiting about 90-comb-like teeth, two rows of vertical teeth about 22, pharyngeal teeth long, and oblong and annulate spermatheca [22]. In the present study, we observed that cibarium teeth of Se. brevicaulis varied in number of posterior teeth and row of anterior teeth; genetically cyt-b showed more than 98% similarity, indicating these two species of specimens from Laos could be varieties of each other. To confirm this observation, further taxonomic studies on a larger scale among SE Asian countries comparing both morphology and genetics of Laotian specimens with Se. brevicaulis from Vietnam and Se. rudnicki from Malaysia, where the original samples were described, is needed.

o Phlebotomus argentipes is colored in green (indicating a sequence generated in this study), but Lines 412–413 state that Ph. argentipes was not recorded, only specimens identified as Ph. argentipes-like. Please clarify this inconsistency.

Response:

Thank you for pointing out this error. This reference sequence of *Phlebotomus argentipes* indeed originated from a study conducted in India. We changed the colour to black.

Description of new sand fly taxa:
I do not feel comfortable commenting on or judging the taxonomic traits used for the new species description.
However, with the advent of molecular tools, I would recommend generating additional reference nucleotide sequences for key genetic markers in new species descriptions. While not mandatory, this is a good practice that can facilitate future discussions and reassessments of species taxonomy. In addition to cyt-b, other mitochondrial markers such as cox1, ND4, ITS1, 16S rRNA, and 12S rRNA might be considered for this and future descriptions.
Response:

We are confident in the morphological characters and the cyt-b gene analysis performed in this study. Nevertheless, we commit to sequencing other genes of this new species and will deposit them afterwards, as we don’t want to further delay the reviewing process.

Leishmania detection:
• Please clarify the criteria for including GenBank sequences.
• A best-hit table with BLAST results could be beneficial for Trypanosoma sp. molecular identification.

Response:

We added and changed lines 369-370 to “BLAST analysis showed that none of the sequences corresponded to Leishmania, but did, however, match with sequences of *Trypanosoma* spp.”

We changed line 376-377 to: “The phylogenetic analysis of ITS1 sequences generated in this work, together with sequences retrieved from BLAST, showed that *Trypanosoma* spp. from *Ch. eunicegalatiae* is a sister group of *Trypanosoma* spp. found from sandflies from southern Thailand namely *Sergentomyia* sp. (OL332783) and *Id. asperulus* (OL332784).”

• Figure 6:
o Ensure that the legend follows the same format as the phlebotomine tree.

Response:

We changed Figure 6: to “Maximum likelihood phylogenetic tree constructed from Trypanosoma ITS1 region. Sequences from BLAST results were selected and included for analysis. The numbers on the branches represent the bootstrap values (%) derived from 1000 replicates. Sequences obtained from this study in Laos are highlighted in red arrows. “n” indicates the number of sequences. Different colors represent host types.”

o Please double-check the description for the sequence OL332783. In the original publication, this sequence is linked to Sergentomyia sp., whereas in your tree, it appears as Id. asperulus. In the main text, you refer to Sergentomyia sp. Please correct this accordingly.
Response:

We corrected to “*Sergentomyia* sp.”.

Blood meal detection:
• Line 354: “A total of 29 semi-gravid females”. However, in Methods (Line 162), it states: “Engorged sand fly samples were used to determine the blood meal origin.” Do the authors consider these terms synonymous? Please revise for consistency.

Response:

We change “Engorged” in line 194 to “Semi-gravid”.
• A positive control is required to confirm that the PCR assay worked as expected.
Response:

We added line 199: “Human blood samples were used as positive controls.”. and we added “Unfortunately, except for positive controls, no samples were successfully amplified, which is likely due to over-digestion of the blood meals” to lines 388-389.
Discussion:
• Lines 363–371: This information is already reported in the Results section.

Response:

We changed lines 369: from “The 25 sandfly species identified included: *Ch. eunicegalatiae, Id. longiforceps, Ph. barguesae, Ph. breyi, Ph. kiangsuensis, Ph. mascomai, Ph. argentipes*-like*, Ph. shadenae, Ph. sinxayarami, Ph. stantoni, Ph.* (*Adlerius*) sp.1, *Se. anodontis, Se. bailyi, Se. barraudi* group*, Se. brevicaulis, Se. dvoraki, Se.* sp.1*, Se. hivernus, Se. khawi, Se. perturbans, Se. phasukae, Se. sutherlandi* n. sp., *Se. sylvatica, Se. tambori,* and *Gr. indica*. ”  to “A total of 25 sandfly species of five genera were identified.”.
• Lines 387–388: What environmental conditions in karstic areas may promote optimal sand fly incidence?

Response:

We add lines 424-426: “Karstic areas, with their humid microclimates, abundant sheltered crevices, organic-rich soils, and presence of vertebrate hosts, may create ideal conditions for this sandfly species.”.
• Lines 445 and 450: Could the authors comment on the species complexes Sergentomyia anodontis s.l. and Sergentomyia bailyi s.l.? What other species could be hidden or misidentified under these nominal species? Could additional genetic markers help distinguish them?

Response:

We added lines 484-485: “Future larger-scale on morphological and molecular studies are clearly needed to further clarify the taxonomy of this probable complex of species. The present…..”.

We added lines: 486-487: “…, this species may represent a complex species group; ….”

Based on our observations during previous studies, these two species are like a species complex group. However, to identify other species that could be hidden under these species, more studies are warranted to 1) re-examine all sample slides recorded in this region for morphological study; 2) as well as to collect more samples from the locations where the specimens of interest were collected for additional molecular analysis.

• Line 460: Is Se. gemmea-like represented by the sequence “Se. sp 1” in the phylogenetic tree? If so, please revise accordingly.

Response:

We added line 498 “(*Se*. sp 1).”.
• Line 469: If any sequence of Se. quatei is available, please include it.

Response:

Unfortunately, we couldn’t find any cyt-b sequence of Se. quatei in GenBank database.

Reviewer #3: I am underwhelmed by the results, but they are interesting. The issue is whether 0.1% positivity is epidemiologically meaningful. Without blood-meal analysis it's impossible for someone else to followup the study. Thus normally the mammals, birds would be trapped and sampled for the tryps - in a followup study. The situation is if I could be certain that sandflies were transmitting tryps thats huge news. However, I cannot be certain it's not incidental feeding, i.e. the tryps will not propagate in the sandfly nor be transmitted. That's the central issue here, there's not enough positives to exclude incidental feeding but equally 0.1% is an epidemiologically acceptable prevalence in a flying vector. Then if we ask the question, well the flies may not be heavily infected but the mammals or birds will have high tryp prevalence; reservoir hosts or tryps can have extreme prevalence of infection. The issue here is we don't know what the sandflies are feeding on, so there's no chance of a follow-up study. Alternatively if the authors had obtained tryp isolates - that would be interesting because they can exist perpetually if carefully cultured and stored.

In summary, strictly from my point of view - excluding the importance of a new species of sandfly - there's not enough here to say this is a PLoS NTD paper. So I will leave this to the entomologists to guidance on the suitability of paper for the journal.

We appreciate the reviewer’s feedback and acknowledgment of needing assistance in understanding the importance of the findings and what they add to the medical literature. Based-upon the opinion of the other reviewers and the improvements made in this latest version of the manuscript, we believe the importance of the results as well as significant conclusion related to improving taxonomy, identification of a new species, defining vector ecology in Laos, and detecting trypanosomes is of significant novelty and importance to warrant publication in the journal.

Reviewer #4: The manuscript by Vongphayloth et al. presents the results of an interesting study describing the sand fly fauna in Laos. The study is important due to the scarcity of information on this issue in the region. Considering the role of some sand fly species in transmitting protozoan parasites such as leishmania, the results contribute new information for the surveillance of this parasitic disease. The findings also presented the description of a new species to science, increasing the knowledge of the diversity of this insect group.

lines 49-53: Studies should be conducted in a wider geographical location with a higher number of traps per area in order to collect a greater number of sand flies to better define this vector and its pathogens. I recommend reviewing this sentence, because collecting a large number of sand flies is not sufficient for vector definition. I suggest modifications to: “Studies should be conducted, increasing the sampling effort to analyze population dynamics and other vector capacity parameters to determine the vector species and pathogen circulation.”

Response:

We changed lines 49-53: from “Sandflies in Laos exhibit significant diversity and distribution across seven provinces, with species composition varying by habitat. Unknown *Trypanosoma* DNA was detected using the Leishmania PCR test. Studies should be conducted in wider geographical location with a higher number of traps per area in order to collect a greater number of sandflies to better define this vector and its pathogens.”

To “This study reveals a notable diversity of sandfly species across seven provinces in Laos, with the highest species richness observed in karstic cave environments. While no *Leishmania* DNA was detected, the unexpected identification of unknown *Trypanosoma* species in *Chinius* *eunicegalatiae* and *Idiophlebotomus* *longiforceps* suggests the potential presence of unrecognized trypanosomatid pathogens in the region. These findings underscore the need for broader geographic surveillance and increased sampling efforts to improve our understanding of sandfly ecology and the pathogens they may carry in Laos.”.
