# Supplementary material for: Analysis of Phlebotomine sandflies in Laos from 2014–2024: Inventory, description of a new species, screening for Leishmania and detection of Trypanosoma
Source: PLoS Negl Trop Dis. 2026 Jan 2;20(1):e0013641. doi: 10.1371/journal.pntd.0013641 (PMC12795457; doi:10.1371/journal.pntd.0013641)
Supplement: Attachment — Submitted filename: Rebuttal_letter20250930.docx [file pntd.0013641.s008.docx]

**Response to Reviewers**

Dear Editors,

Please find below our response to reviewers, other changes were also added directly in the manuscript.

Best regards,

Khamsing Vongphayloth

**Reviewers' comments:**

Reviewer's Responses to Questions

**Key Review Criteria Required for Acceptance?**

As you describe the new analyses required for acceptance, please consider the following:

**Methods**

-Are the objectives of the study clearly articulated with a clear testable hypothesis stated?

-Is the study design appropriate to address the stated objectives?

-Is the population clearly described and appropriate for the hypothesis being tested?

-Is the sample size sufficient to ensure adequate power to address the hypothesis being tested?

-Were correct statistical analysis used to support conclusions?

-Are there concerns about ethical or regulatory requirements being met?

Reviewer #2: The methods were performed to answer the objectives of the study.

Reviewer #3: The authors have done a reasonable job of their tryp trees; specifically they were asked to get a new representative sample distribution so a "tryp specialist" can interpret what type of tryps the authors have detected. However, the phylogenetic methods need a far more detailed explanation. I would recommend the commands they used are presented in the appendix. Furthermore, the alignment has not been manually inspected. Manual curation and certification is essential and was not described. Therefore I would strongly suggest this is performed and where necessary the trees re-calculated. This applies to both sandfly and tryp trees. The congruence between MEGA and IQTREE needs reporting and BOTH sets of bootstraps should be represented on the trees. It's not clear which tree, nor which bootstraps the authors have presented.

Response: we realigned our dataset with different alignment algorithms and added details on the technical analysis of the *Trypanosoma* phylogenetic tree are provided in S1 Appendix. The phylogenetic tree analysis methods were revised from lines 217 – 231.

**Results**

-Does the analysis presented match the analysis plan?

-Are the results clearly and completely presented?

-Are the figures (Tables, Images) of sufficient quality for clarity?

Reviewer #2: The authors have followed the reviewers' recommendations. The current version if the manuscript has been improved.

Reviewer #3: The key criticism is that the trees have substantially below publication, moreover the tryp tree has key information which I am unable to read. Specifically I want to know which tryps the authors sequences form sister groups with, i.e. which insects were the database sequences recovered. The key question is whether these are incidental infections or an epidemiology (between sandflies and tryps) which has yet to be formalised. The sister group insect vector of the authors sequences will give an indication. There is no option but for the authors to perform a new revision and return this for inspection, because the tree is not intelligible.
Secondly, the authors have poorly described the location within the tree of their tryp sequences. T. sp. isn't a tryp. Again we are looking for overall positioning, i.e. rodent versus T. rangeli/ T. cruzi clades and other information concerning vector/vertebrate isolation data. This needs to be professionally described within the results section and it significantly below the required standard. Again, what we're assessing is whether their is evidence supporting or rejecting incidental infection or new epidemiological cycle.

PLoS NTD is an internationally recognised journal of high standing and half-baked descriptors are not acceptable in this journal.

Response: we found that there was high sequence divergence among ITS1+5.8S rRNA sequences generated from this study and other known ITS1+5.8S rRNA sequences of *Trypanosoma* available from GenBank database across MAFFT, MUSCLE, and CLUSTALW alignments. We added S1 Appendix Fig. C–E for manual inspection. So, from this dataset, it is not possible to position the *Trypanosoma* that we detected to other known *Trypanosoma*. We also provided S1 Appendix Table A for more detailed metadata of ITS1+5.8S rRNA sequences available from GenBank and used in this analysis. Other gene markers will be needed to confirm this observation (see more in the discussion part). Different tree topologies and different bootstrap values from different alignments, different software for tree builds were shown separately in S1 Appendix Fig. F–H.

Changes were made from Line 379 to 395

**Conclusions**

-Are the conclusions supported by the data presented?

-Are the limitations of analysis clearly described?

-Do the authors discuss how these data can be helpful to advance our understanding of the topic under study?

-Is public health relevance addressed?

Reviewer #2: Yes.

Reviewer #3: The discussion has been handled nicely in identifying the weaknesses/limitations of the study. The authors have omitted citations by Stevens/Gibson (tryps) and Ready (sandflies). This should be addressed; was their tree congruent with the phylogenies of Stevens/Hamilton/Gibson? Furthermore although Ready focused on sandflies in north African counties (possibly the Middle East) this investigator was an early exponent of cyt B taxonomic identification and population genetics. These issues require addressing in the discussion. The citation for Dvorak was appropriate.

Response:

1. For sandfly, we added lines 465 – 468: “Our *cyt-b* phylogeny is congruent with earlier work [27], which demonstrated the value of mitochondrial haplotype analysis for resolving taxonomic relationships and population structure in *Phlebotomus* subgenera such as *Phlebotomus* and *Larroussius*. This supports its broader applicability for distinguishing closely related species, including *Adlerius* spp. [45].”
2. For *Trypanosoma*, we added more discussion on the high divergence of our sequences and of our results with Stevens and Gibson, 1999; Khositharattanakool P., et al., 2024. We revised and made changes in lines 533 to 557.

**Editorial and Data Presentation Modifications?**

Use this section for editorial suggestions as well as relatively minor modifications of existing data that would enhance clarity. If the only modifications needed are minor and/or editorial, you may wish to recommend “Minor Revision” or “Accept”.

Reviewer #2: Line 64: Since there is no information about this Trypanosoma spp. pathogenicity, I recommend deleting “pathogens.”

Response: “pathogens” was deleted

Line 77: Please italicize Leishmania, and include “parasites” (i.e., Leishmania parasites).

Response: “*Leishmania* parasites”

Lines 138–148 (Fig 1): Please ensure to mention that the red stars refer to the collection sites.

Response: Added “Red stars represent the collection sites in this study.”

Line 180: “spp.” should not be italicized.

Response: changed

Line 198: Please cite the origin of the human blood source. Was the DNA extraction method used the same as for sand flies? Please note that mentioning “human blood” may raise ethical concerns. If the authors used a DNA sample from a previous study, I suggest mentioning that instead, along with the origin of the material.

Response: changed from “Human blood samples” to “DNA was extracted as previously described [32], and used as positive controls”

Lines 205–206: Please cite the origin of L. major and L. tropica DNA (e.g., cultured strain + strain number).

Response: added reference [35]

Line 226: Please insert a comma after "508 males."

Response: inserted “,” after "508 males.".

Tables 2 and 3: Please also include the abbreviations of genera in the notes.

Response: added the abbreviations

Line 511: “Complex species group” should be corrected to “species group” or “species complex.” Both terms are used to refer to informal assemblages of taxa, particularly among phlebotomine sand flies (PMID: 33480064; DOI: 10.1186/s13071-025-06748-5).

Response: deleted “complex”

Lines 514-517: “However, after more recent studies, species reported in this region may include Se. hivernus, Se. dvoraki, Se. khawi, and Se. gemmea [20]. However, the records of Se. gemmea in Laos are pending revision, as the pairwise distance of the cyt-b gene between specimens from Laos and Thailand was only 0.06 (6%) [14].”
This is a bit confusing. Do the authors consider the pairwise distance of 6% too low? As written, it sounds like the molecular distance of 6% might indicate different species (Laos vs. Thailand), but the morphological difference observed did not meet the criteria used by the authors for species description.

Response: deleted “only”. We consider the pairwise distance of 6% too high.

Line 519: Please consider replacing “to have” with “to exhibit” and “needed” with “necessary.”

Response: changed

Line 519: For better reading flow, I suggest splitting this sentence: “…for a new species. Instead, we will await…”

Response: changed

Line 532: Please use “Southeast Asia” consistently throughout the text for uniformity.

Response: changed

Reviewer #3: What has been presented by the authors is reasonably technically sound but significantly below acceptable standard in presentation and interpretation.

Response: Thank you for your comments. We tried to improve this revised version.
